# Abdominelle Weichgewebstumoren

**DOI:** 10.1007/s00292-022-01128-7

**Published:** 2022-10-12

**Authors:** Eva Wardelmann, Anna Kuntze, Marcel Trautmann, Wolfgang Hartmann

**Affiliations:** Gerhard-Domagk-Institut für Pathologie, Universitätsklinikum Münster, Albert-Schweitzer-Campus 1, Gebäude D17, 48149 Münster, Deutschland

**Keywords:** Weichgewebstumoren, GIST, Molekulare Klassifikation, Translokationspositive Neoplasien, Referenzpathologie, Soft tissue tumors, GIST, Molecular classification, Translocation-positive neoplasias, Reference pathology

## Abstract

Gastrointestinale Stromatumoren sind mit einer Inzidenz von 10–15 Fällen pro 1 Mio. Einwohner in Deutschland die häufigsten mesenchymalen Tumoren im Abdominalbereich. Ihre eindeutige Identifikation und Charakterisierung ist für betroffene Personen prognostisch und therapeutisch von großer Bedeutung. Ebenso wichtig ist aber auch die klare Abgrenzung anderer mesenchymaler Neoplasien, zu denen leiomyomatöse, neurogene, adipozytäre und fibroblastäre Tumoren gehören. Zudem wächst die Zahl der translokationspositiven Entitäten, die sich nur mit entsprechenden molekularen Methoden eindeutig diagnostizieren lassen. Ziel dieses Beitrags ist es, für deren sichere Identifikation praktische Hinweise zu geben. Eine mögliche Referenzpathologie kann die Diagnosefindung unterstützen.

## Abstract (en)

Gastrointestinal stromal tumors are the most common mesenchymal tumors in the abdomen and occur in Germany with an incidence of 10 to 15 cases per million inhabitants. Clear identification and characterization are of major importance for the prognosis and therapy of patients. Similarly, they have to be differentiated from other mesenchymal neoplasias such as leiomyomatous, neurogenic, adipocytic, and fibroblastic tumors. Additionally, the number of translocation positive neoplasias is increasing, requiring the use of adequate molecular assays. The aim of this paper is to give practical advice for their identification. Reference pathology is one possibility to support the correct diagnosis.

Abdominelle Weichgewebstumoren stellen sowohl im Biopsiegut als auch in Operationspräparaten aufgrund ihrer Vielgestaltigkeit eine diagnostische Herausforderung dar. Während gastrointestinale Stromatumoren als häufigste mesenchymale Tumoren in dieser Region aufgrund definierter immunhistochemischer Marker zumeist gut zu identifizieren sind, bereiten die zahlreichen Differenzialdiagnosen aufgrund ihrer Seltenheit durchaus Schwierigkeiten und erfordern nicht selten den Einsatz von Molekularpathologie. Die wichtigsten werden hier dargestellt und sollen den Zugang zu diesen Entitäten erleichtern.

## Hintergrund

Das Spektrum der im Gastrointestinaltrakt anzutreffenden Weichgewebstumoren ist breit. Zweifelsohne am häufigsten kommen gastrointestinale Stromatumoren (GIST) vor. Da diese Tumorentität ein sehr variables biologisches Verhalten aufweist und zugleich eine zumeist hochgradig effektive Therapie mit Tyrosinkinaseinhibitoren zur Verfügung steht, ist es umso wichtiger, GIST zum einen eindeutig zu identifizieren und zu typisieren und zum anderen wichtige Differenzialdiagnosen sicher auszuschließen. Die HE-Morphologie ist ein wichtiger erster Schritt, um eine entsprechend zielführende Immunhistochemie und ggf. auch Molekularpathologie anschließen zu können. Es folgen die wichtigsten abdominellen Weichgewebstumoren mit besonderem Augenmerk auf die diagnostischen Kriterien sowie die ganz unterschiedlichen Biologien der verschiedenen Entitäten.

## Gastrointestinale Stromatumoren

**Figure 1:**
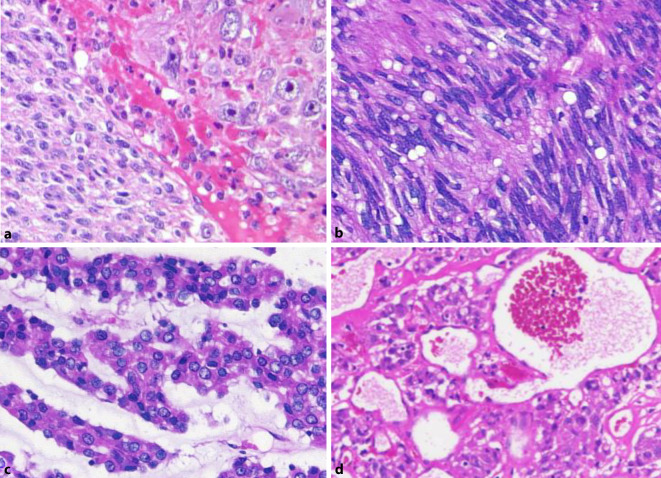

**Figure 2:**
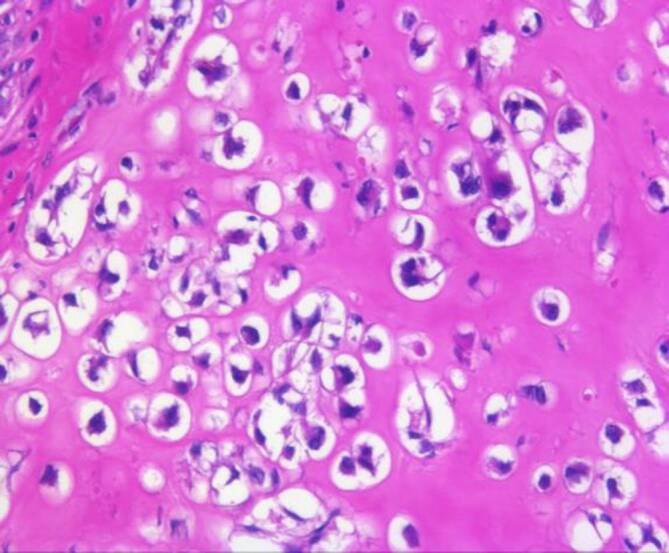

**Figure 3:**
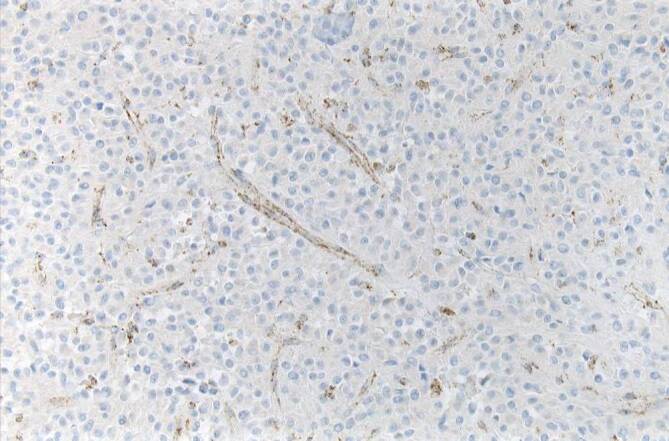

Gastrointestinale Stromatumoren sind die häufigsten mesenchymalen Tumoren im Abdomen. Sie werden mehrheitlich im Magen (ca. 60 %) gefolgt vom Dünndarm (25–30 %) sowie dem Rektum (ca. 5 %) und am seltensten im Ösophagus (< 1 %) beobachtet. Zudem kommen selten auch Tumoren ohne direkten Bezug zum tubulären Gastrointestinaltrakt (sog. E‑GIST) vor, deren Anteil wahrscheinlich unter 5 % liegt [1]. Die meisten GIST sind durch eine gezielte Immunhistochemie problemlos zu diagnostizieren. Ein in unseren Händen bewährtes Antikörperpanel besteht aus CD34, CD117, DOG1, SDHB und Ki67. Schwierigkeiten bestehen gelegentlich bei ungewöhnlicher Histomorphologie, dies gilt insbesondere für epitheloide, pseudoangiomatöse oder auch zystisch-regressive Tumoren (Abb. 1). In histomorphologisch herausfordernden Fällen kann die Diagnose molekularpathologisch gesichert werden, da ca. 85 % der GIST aktivierende Mutationen in den Rezeptortyrosinkinasen *KIT* oder *PDGF-Rezeptor alpha* (*PDGFRA*) aufweisen. Gerade bei Mutationen im letztgenannten Gen werden gehäuft ungewöhnliche Phänotypen, teilweise auch mit erheblicher Reduktion oder gar vollständigem Fehlen einer KIT-Rezeptor-Expression beobachtet, zudem weisen diese Tumoren häufig mehrkernige Riesenzellen („flower cells“) und eine hyaline oder chondroide Matrix auf ([2]; Abb. 2). Neben der diagnostischen Relevanz der Molekularpathologie ist diese auch prognostisch und prädiktiv von Bedeutung. Deletionen im besonders häufig betroffenen Exon 11 des *KIT*-Gens gehen überzufällig häufig mit einem aggressiven klinischen Verlauf einher [3], während die fast ausnahmslos im Magen lokalisierten GIST mit *PDGFRA*-Mutation mehrheitlich eine günstigere Biologie zeigen. Zugleich sprechen gerade die *KIT-*Exon-11-mutierten GIST besonders gut auf den oralen Tyrosinkinaseinhibitor Imatinib an, dessen Einsatz sowohl neoadjuvant als auch adjuvant und in der metastasierten Situation den Goldstandard darstellt [4]. Der häufigste Mutationstyp im *PDGFRA* in Exon 18 (p.D842V) führt hingegen zu einer primären Resistenz gegenüber Imatinib. Erst kürzlich konnte für diesen spezifischen Mutationstyp mit Avapritinib eine wirksame Substanz zugelassen werden [5]. Im Krankheitsverlauf wird aber die Behandlung von GIST nicht selten durch das Auftreten von Sekundärmutationen verkompliziert, sodass weitere Therapiestrategien entwickelt werden müssen [6]. Sehr selten werden *KIT/PDGFRA*-Mutationen in der Keimbahn gefunden, die bei *KIT* das Auftreten von multiplen GIST und systemischen Mastozytosen zur Folge haben können, während beim PDGFRA-Syndrom neben GIST inflammatorische fibroide Polypen, Fibrolipome und übergroße Hände beobachtet werden [7]. Bis heute sind etwas mehr als 50 Familien mit einem PDGFRA- oder KIT-Syndrom beschrieben. GIST ohne *KIT*- oder *PDGFRA*-Mutation (früher als sog. Wildtyp-GIST bezeichnet) machen etwa 10–15 % aller Fälle aus [3] und lassen sich in die Gruppe der Tumoren mit bzw. ohne Succinatdehydrogenase(SDH)-Defizienz aufteilen [8, 9]. Der SDH-Komplex ist Bestandteil des Zitratzyklus und membranständig in den Mitochondrien lokalisiert. Er besteht aus den 4 Untereinheiten SDHA, SDHB, SDHC und SDHD und katalysiert die Oxidation von Succinat zu Fumarat. Bei einem Defekt einer der Untereinheiten kommt es unabhängig von der betroffenen Untereinheit zu einem immunhistochemischen Ausfall von SDHB (Abb. 3), weshalb dieser Marker zu unserem diagnostischen Basispanel gastrointestinaler Stromatumoren gehört. Wenn SDHB exprimiert wird und der SDH-Komplex somit intakt ist, liegt den Tumoren zumeist eine *BRAF*-Mutation (bislang ausschließlich p.V600E, [10]) zugrunde, fast alle übrigen Tumoren zeigen eine Aktivierung des RAS-RAF-Signalwegs bedingt durch *NF1*-Mutationen. Diese gehen zumeist mit einer assoziierten Neurofibromatose Typ 1 einher, seltener liegt ein sporadisches Auftreten der Mutation im Tumor vor. Die deutlich größere Gruppe der non-*KIT*/non-*PDGFRA*-mutierten Tumoren sind die GIST mit SDH-Defizienz, wobei auch hier eine familiäre Häufung zu beobachten ist. Bei Keimbahnmutation in einem der 4 SDH-Komplexpartner (SDHA, SDHB, SDHC oder SDHD) tritt das Carney-Stratakis-Syndrom auf [11, 12], das synchron oder metachron mit Paragangliomen einhergeht. Deutlich seltener kommen *SDH*-Mutationen auch in sporadischen GIST vor [13]. Im Falle einer epigenetischen Regulationsstörung mit SDHC-Ausfall [14] wird die Carney-Triade beobachtet, bei der die Betroffenen neben Paragangliomen auch pulmonale Chondrome entwickeln. Genetisch wenig verstanden sind bislang sporadisch auftretende GIST vom sog. pädiatrischen Typ, die bevorzugt, jedoch nicht ausschließlich im Kindesalter auftreten und keine der bislang bekannten Alterationen aufweisen [15].

## Wichtige Differenzialdiagnosen von GIST

Die Liste der Differenzialdiagnosen von mesenchymalen Tumoren im Abdominalbereich ist lang. In der ganz überwiegenden Mehrzahl der Fälle gelingt die Differenzialdiagnose durch Einsatz der richtigen immunhistochemischen Marker, ggf. ist eine ergänzende molekulare Diagnostik erforderlich. Durch den zunehmenden Einsatz von RNA-Sequenziermethoden und die Tatsache, dass gerade monomorphzellige mesenchymale Neoplasien häufig mit Translokationen assoziiert sind, wächst die Anzahl der neu beschriebenen Entitäten erheblich. Es wird hiervon eine Auswahl vorgestellt, wobei aufgrund der Aktualität einige Subtypen noch nicht in die neue WHO-Klassifikation [16] aufgenommen wurden.

### Leiomyom

Leiomyome im Gastrointestinaltrakt finden sich am häufigsten am gastroösophagealen Übergang und im proximalen Magen, in dieser speziellen Lokalisation sind sie sogar häufiger als GIST anzutreffen. Einen Sondertyp stellt das Leiomyom der Lamina muscularis mucosae im Colon dar, das sich zumeist als kleiner submuköser Polyp mit typischer leiomyomatöser Histomorphologie präsentiert, während GIST im Colon (mit Ausnahme des Rektums) exzeptionell selten sind. Leiomyome in diesem Organsystem sind immer gutartig und häufig hypozellulär. Im Gegensatz zu Leiomyomen anderer Lokalisationen sind diese im Gastrointestinaltrakt typischerweise Vimentin-negativ und kräftig positiv für glattmuskuläres Aktin, Calponin, Caldesmon und Desmin. Schwierigkeiten bereitet ihre Diagnose gelegentlich im bioptischen Setting, da der hohe Gehalt an CD117-positiven Mastzellen und DOG1-positiven interstitiellen Zellen einen GIST imitieren kann, der allerdings nicht eine derartig kräftige Positivität für Desmin aufweisen sollte und zudem Vimentin-positiv ist.

### Leiomyosarkom

**Figure 4:**
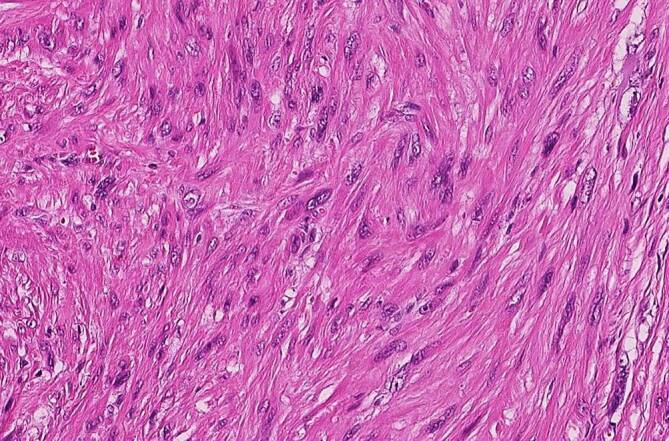

Leiomyosarkome treten bevorzugt im Colon und Retroperitoneum auf und sind zumeist durch erhebliche zelluläre Pleomorphien gekennzeichnet, sodass ihr Einordnung als maligner Tumor nicht schwerfallen sollte (Abb. 4). Mitosen – auch atypische – sind leicht zu finden. Immunhistochemisch ist eine Expression von zumindest 2 glattmuskulären Markern erforderlich, wobei einer davon Desmin sein sollte. Außerdem sollte eine leiomyosarkomatöse Komponente eines dedifferenzierten Liposarkoms durch Nachweis bzw. Ausschluss einer *MDM2*-Cluster-Amplifikation ausgeschlossen werden [17].

### Schwannom

**Figure 5:**
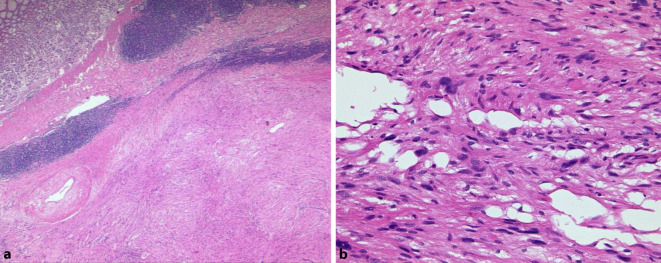

Schwannome des Gastrointestinaltraktes sind gutartig und kommen bevorzugt in der Magenwand, gelegentlich aber auch im Dünndarm vor. Ganz charakteristisch sind wallartig angeordnete dichte lymphatische Infiltrate an der äußeren Zirkumferenz, gelegentlich auch innerhalb der Läsion (Abb. 5). Einzelzellpleomorphien sind nicht selten anzutreffen und ein Zeichen der Degeneration. Dies lässt sich zumeist bei Einsatz des Ki67-Antikörpers gut verifizieren, da diese Zellen nicht proliferieren. Die Diagnose als Schwannom wird dadurch erschwert, dass keine Antoni-A- oder Antoni-B-Muster auftreten und Regressionserscheinungen den Phänotyp stark verschleiern können. Bei Einsatz von Antikörpern gegen S100-Protein und Sox10 stellt die Diagnose aber im Allgemeinen keine Schwierigkeit dar. Eine Abgrenzung vom deutlich selteneren Neurofibrom gelingt durch Einsatz von CD34, das in diesen kräftig koexprimiert wird.

### Maligner peripherer Nervenscheidentumor (MPNST)

Der MPNST stellt im Abdominalbereich mit Bezug zum Gastrointestinaltrakt eine Rarität dar. Häufiger finden sich diese Läsionen im Retroperitoneum, nicht selten mit Bezug zu größeren Nerven. Nach einer zugrunde liegenden NF1-Erkrankung sollte klinischerseits gefahndet werden. Phänotypisch ähneln MPNST analogen Tumoren anderer Primärlokalisation und sind somit oft negativ für S100 und SOX10, ferner ist ein Ausfall von H3K27me3 festzustellen.

### Inflammatorischer fibroider Polyp (IFP)

**Figure 6:**
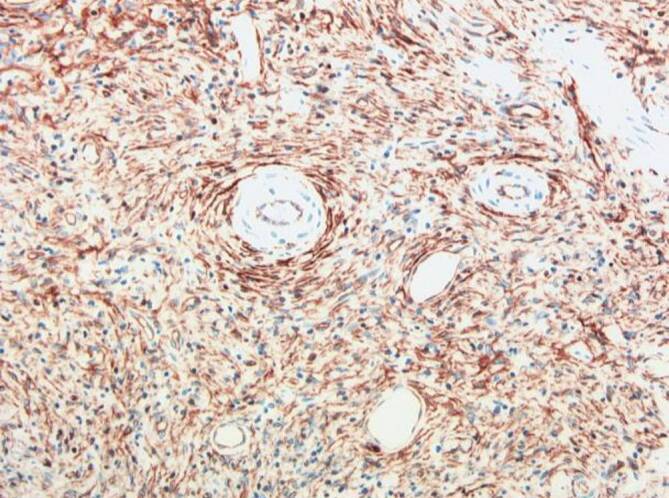

IFP sind grundsätzlich gutartig und führen gerade im Dünndarm nicht selten zu Invaginationen mit entsprechender akuter chirurgischer Notfallsituation. IFP kommen sowohl im Magen als auch im Dünndarm vor und können histomorphologisch vielgestaltig sein. Zumeist wird CD34 exprimiert, dieser Marker kann jedoch auch vollständig fehlen. In einem oft lockeren Fibroblastenproliferat finden sich in wechselnder Dichte gemischte inflammatorische Infiltrate mit wechselndem Eosinophilengehalt, die Fibroblasten sind nicht selten zwiebelschalenartig um Gefäße herum angeordnet (Abb. 6). Molekularpathologisch finden sich häufig *PDGFRA*-Mutationen, die ganz analog auch in GIST vorkommen, wenngleich die Mutationstypen sich durchaus unterscheiden [18, 19]. Im Gegensatz zu GIST exprimieren IFP weder CD117 noch DOG1. Seltene syndromale Formen mit synchronem Auftreten von IFP und GIST sind kasuistisch beschrieben [7].

### Desmoidfibromatose

**Figure 7:**
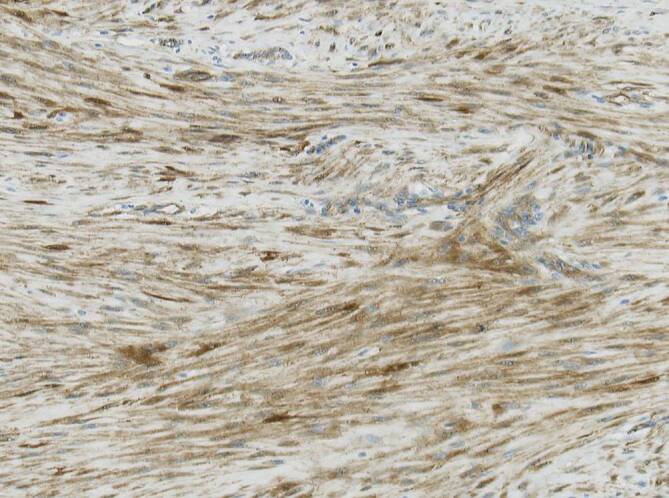

Die abdominelle Fibromatose ist der Gruppe der Tumoren mit intermediärer Biologie (lokal aggressives Wachstum) zuzuordnen. Bei Bezug zum Gastrointestinaltrakt liegen häufiger scharf begrenzte kugelige Tumoren vor, die makroskopisch mit GIST verwechselt werden können. Dabei wächst der Tumor mikroskopisch oft infiltrativ ins Fettgewebe und örtliche Strukturen ein. Histomorphologisch zeigt sich ein typisches faszikuläres Erscheinungsbild aus fibroblastischen Zellen mit spitzzipflig ausgezogenen Zellkernen, manchmal imponieren die Zellen auch triangulär. Charakteristisch ist eine nukleäre Expression von β‑Catenin (Abb. 7), der in mehr als 70 % der Fälle eine *CTNNB1*-Mutation zugrunde liegt [20]. Deutlich seltener werden diese Tumoren im Kontext eines Gardner-Syndroms (in Kombination mit der familiären adenomatösen Polypose) beobachtet, bei dem Keimbahnmutationen im *APC*-Gen ursächlich sind [21]. Im Gegensatz zu einer bis vor wenigen Jahren üblichen radikalen operativen Prozedere mit bisweilen hohen Rezidivraten wird heute überwiegend eine Watch-and-Wait-Strategie favorisiert, wobei in einem Teil der Fälle auch spontane Tumorregressionen beobachtet werden [22].

### Solitärer fibröser Tumor (SFT)

**Figure 8:**
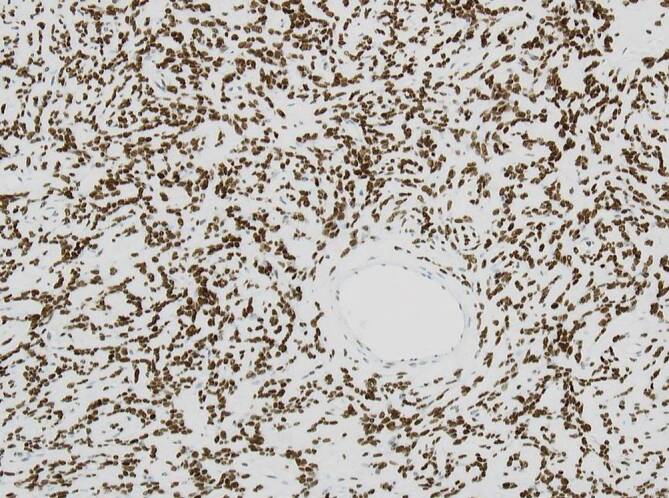

Solitäre fibröse Tumoren kommen im gesamten Körper und somit auch im Gastrointestinaltrakt bzw. Abdominalbereich vor. Die Läsionen können eine beträchtliche Größe erreichen und gerade im Abdomen lange unbemerkt bleiben. Mit zunehmender Größe steigt das Risiko einer Metastasierung. Insgesamt werden diese Läsionen als intermediär maligne mit seltener Metastasierung eingeordnet. In dem aktuell in der WHO-Klassifikation von 2020 publizierten Kapitel wird zur Einschätzung der biologischen Wertigkeit ein Score verwendet, in den Alter, Tumorgröße, Mitosezahl und Nekrose einfließen [16]. Der SFT ist zumeist CD34-positiv und exprimiert zudem CD99 und bcl2. Charakteristisch sind hämangioperizytomartig gewinkelte, oft englumige, nicht selten wandhyalinisierte Gefäßstrukturen und zwischenliegend isomorphzellige Fibroblastenproliferate ohne spezifisches Wuchsmuster. Die Tumoren können zystisch transformieren oder auch kräftig regressiv hyalinisieren, oft mit abruptem Wechsel zwischen verschiedenen Morphologien. Diagnostisch sehr wertvoll ist der Antikörper gegen STAT6, der zu einem kräftigen nukleären Färbesignal führt und so verlässlich ist, dass zumeist auf eine weitere molekulare Typisierung verzichtet werden kann (Abb. 8). SFT tragen typischerweise *STAT6-NAB2*-Fusionstranskripte.

### Perivaskulärer epitheloidzelliger Tumor (PECom)

Auch PECome können im gesamten Organismus und somit auch abdominell vorkommen. Hier verursachen sie differenzialdiagnostische Schwierigkeiten, da sie z. B. ebenfalls den KIT-Rezeptor exprimieren können. Charakteristisch ist eine myomelanozytäre Differenzierung mit Expression von glattmuskulärem Aktin und melanozytären Markern wie HMB45 und MelanA. Die Tumoren treten assoziiert mit *TSC2*-Mutationen auf, eine kleinere Subgruppe weist stattdessen *TFE3*-Translokationen auf [23]. In der letztgenannten Gruppe kann immunhistochemisch TFE3 nachgewiesen werden, auch wenn der Antikörper ein relativ breites Spektrum von Tumoren anfärbt und zu unspezifischen Färbeprodukten neigt.

### Angiosarkom (ASA)

**Figure 9:**
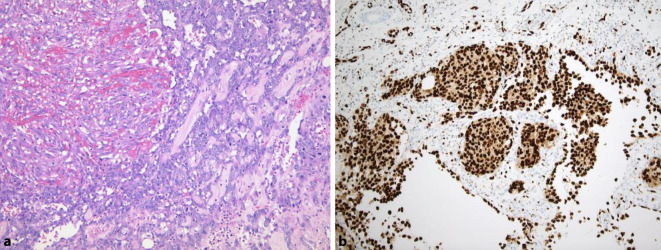

Angiosarkome kommen ebenfalls in jedem Organsystem vor und sind unabhängig von ihrer Lokalisation fast immer mit einer sehr ungünstigen Prognose verbunden. Im Gastrointestinaltrakt tritt als Sonderform außerdem das Kaposi-Sarkom auf, welches sich durch Einsatz einer HHV8-Immunhistochemie gut identifizieren lässt. Angiosarkome sind nicht immer schon morphologisch klar als vasoformativ erkennbar, sodass im Zweifel der Einsatz mehrerer endothelialer Marker in der differenzialdiagnostischen Bewertung erfolgen sollte. Eine alleinige Färbung für CD31 oder CD34 reicht dabei nicht aus. Es sollte auch ein nukleärer Endothelmarker, am besten ERG (Abb. 9), eingesetzt werden [24]. Sekundär auftretende Angiosarkome nach Bestrahlung zeigen nicht selten *c‑MYC*-Amplifikationen [25]. Die c‑MYC-Überexpression kann auch immunhistochemisch detektiert werden [10]. Zudem sind verschiedene molekulare Aberrationen in Angiosarkomen beschrieben, die derzeit aber keine diagnostische oder therapeutische Relevanz besitzen.

### Dedifferenziertes Liposarkom (DDLS)

Das DDLS kommt sowohl im Peritoneum als auch in der Darmwand vor und kann transmural bis in die Schleimhaut des tubulären Gastrointestinaltrakts vorwachsen. Somit kann sich ein solcher Tumor auch in einer endoskopisch gewonnenen Biopsie finden. Bei spindelzelligen Läsionen im Abdominalbereich auch ohne lipomatöse Komponente muss ein DDLS stets am besten mittels Fluoreszenz-in-situ-Hybridisierung für *MDM2* ausgeschlossen werden [26]. DDLS können in verschiedenste Richtungen heterolog differenzieren, sodass Knochen, aber auch Knorpel oder Muskulatur sowie ihre malignen Äquivalente innerhalb der Läsion nachgewiesen werden können. Nicht selten zeigt jede Probe in einem großen Tumor histologisch einen anderen Phänotyp. Die *MDM2*-FISH-Analytik mit Nachweis von Clusteramplifikaten hat sich als sehr hilfreich erwiesen, wenn andere *MDM2*-amplizierte Tumorentitäten konsequent ausgeschlossen werden (wie z. B. das Intimasarkom oder in Knochennähe das Osteosarkom).

### *SWI/SNF*-defiziente Neoplasien

Der *SWI/SNF-*Komplex fungiert als Tumorsuppressor und zeigt in zahlreichen Neoplasien Defekte in einem der multiplen Komplexpartner. Besonders häufig führen diese zu einem Ausfall von SMARCA4 (BRG1) oder SMARCB1 (INI1), die über Helikase- und ATPase-Aktivität transkriptionell Gene regulieren und das Chromatinremodeling beeinflussen. Auf die zahlreichen Entitäten mit einem derartigen Ausfall soll hier nicht eingegangen werden. Jedoch lohnt es sich, bei einem unklaren abdominellen Tumor nach einem INI1- oder BRG1-Ausfall immunhistochemisch zu fahnden. Eine Übersicht findet sich z. B. bei Agaimy [27].

### *ACTB/MALAT1/PTCH1-GLI1*-translozierte Tumoren

Die Gruppe *ACTB/MALAT1/PTCH1-GLI1*-translozierten Tumoren ist durch eine pathologische Aktivierung von GLI1 („glioma-associated oncogene homologe 1“) gekennzeichnet, wobei das *GLI1*-Gen jeweils mit unterschiedlichen anderen Genen eine Fusion bildet. Physiologisch wird GLI1, ein Mitglied der Kruppel-Familie von Zinkfingerproteinen, bei Erwachsenen lediglich im Tubenepithel, im Myometrium und im Hoden exprimiert.

#### *GLI1*-*ACTB1*-Tumor (ehemals Perizytom)

Die Erstbeschreibung einer charakteristischen Translokation t(7;12) erfolgte im Jahr 2004 in Aktin-positiven, perivaskulären myoiden Tumoren der Zunge, des Magens und des Knochens [28]. Fusionspartner von *GLI1 *war *ACTB1* („beta-actin gene“), die Tumoren wurden als Perizytome bezeichnet. Typischerweise treten diese Tumoren bei jungen Frauen unter 40 Jahren auf und zeigen ein gutartiges biologisches Verhalten. Histologisch handelt es sich um epitheloide monomorphe Tumorzellen mit insulärem oder trabekulärem Wuchsmuster. Eine Angioinvasion ist möglich. Immunhistochemisch sind Aktin, gelegentlich Desmin und variabel CD10 nachzuweisen, hingegen kommen KIT, DOG1, CD34, EMA und S100-Protein nicht vor.

#### Plexiformes Fibromyxom

**Figure 10:**
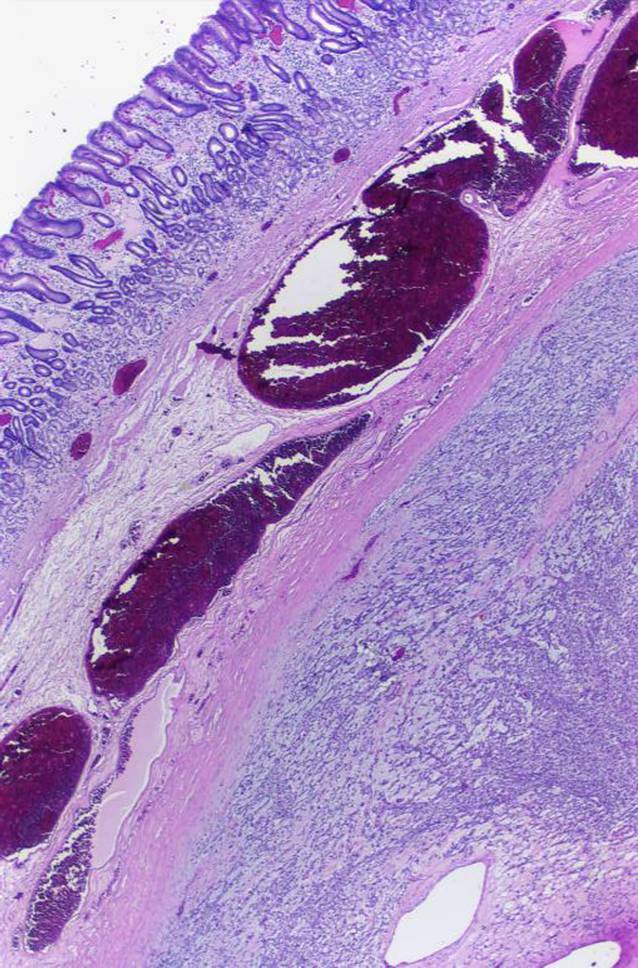

Von Miettinen et al. [29] folgte 2009 eine Fallsammlung von 12 sehr ähnlichen Tumoren im pylorusnahen Magenantrum und deren Bezeichnung als plexiformes Fibromyxom. Eine Übersicht zu weiteren Fallbeschreibungen findet sich bei Spans et al. [30]. Diese kommen fast ausschließlich im Magen vor, sehr seltene Fälle sind in anderen Regionen des tubulären Gastrointestinaltrakts beschrieben. Die Tumoren wachsen multinodulär und weisen eine myxoide, fibromyxoide oder fibröse Matrix mit Einschluss fibroblastischer Spindelzellen auf (Abb. 10). Immunhistochemisch werden Aktin, gelegentlich Desmin und variabel CD10 exprimiert bei Negativität für KIT, DOG1, CD34, EMA und S100-Protein. Selbst bei Nachweis eines angioinvasiven Wachstums verhalten sich diese Tumoren durchweg gutartig. Molekularpathologisch sind eine *GLI1-MALAT1*(„metastasis-associated lung adenocarcinoma transcript 1“)-Fusion und *GLI1*-Polysomien typisch.

#### Gastroblastom

Analoge *GLI1-MALAT1*-Translokationen kommen im ausschließlich gastral beschriebenen Gastroblastom vor [31], das erstmalig 2009 ebenfalls von Miettinen et al. [32] beschrieben wurde und vor allem bei jungen Erwachsenen auftritt. Die biphasischen Tumoren wachsen in Nestern und Rosetten im Wechsel mit einer blanden spindelzelligen, mitunter faszikulären Zellpopulation (Abb. 10). Im Gegensatz zum plexiformen Fibromyxom können Gastroblastome metastasieren. Die Tumoren sind positiv für Zytokeratine, CD56 und GLI1. Differenzialdiagnostisch ist das Synovialsarkom abzugrenzen, was aber bei Einsatz der entsprechenden immunhistochemischen Marker und einer RNA-Sequenzierung problemlos möglich ist.

#### Maligne epitheloide Neoplasie mit *GLI1*-Fusion

Eine weitere Gruppe von Tumoren mit *GLI1*-Fusionen zeichnet sich durch einen anderen immunhistochemischen Phänotyp aus, nämlich durch eine Positivität für S100-Protein, SOX10 und CD56 bei Negativität für SMA, CD117 und DOG1. Histologisch findet sich ein trabekuläres Wuchsmuster, das an Glomustumoren erinnert. Ein biphasischer Aspekt wie bei Gastroblastomen ist nicht zu beobachten. Diese bei jungen Erwachsenen im Weichgewebe auftretenden Tumoren können bis zu 15 cm groß werden und metastasieren. Fusionspartner von *GLI1* sind *ACTB, MALAT1* oder *PTCH1 *[33]. Alternativ wurden ähnliche Tumoren auch mit *GLI1*-Amplifikationen beschrieben, mehrheitlich in Kombination mit *CDK4-* und *MDM2*-Amplifikationen. Alle 3 Gene sind eng benachbart auf dem langen Arm von Chromosom 12 lokalisiert und daher nicht selten koamplifiziert. Die Biologie ist ebenso variabel wie der immunhistochemische Phänotyp [34].

## Fazit für die Praxis

- Gastrointestinale Stromatumoren stellen die häufigsten mesenchymalen Neoplasien im Abdomen dar und können immunhistochemisch zumeist leicht diagnostiziert werden. Ihre molekulare Subtypisierung ist diagnostisch, prognostisch und therapeutisch relevant und sollte bei der Erstdiagnose erfolgen.
- Die aktuelle WHO-Klassifikation hat diverse neue Tumorentitäten aufgenommen, die vor allem durch neu detektierte genomische Alterationen gekennzeichnet sind. Einige Entitäten sind aufgrund der stetig wachsenden Zahl neuer definierender, zumeist molekularer Charakteristika jedoch darin noch nicht zu finden.
- Für eine adäquate Sarkomklassifikation ist das Vorhalten von verschiedenen molekularpathologischen Methoden unerlässlich mit einem wachsenden Stellenwert der RNA-Sequenzierung. Die Referenzpathologie hilft aufgrund der Seltenheit diverser Subtypen bei der Einordnung.
- Die Detektion der verschiedenen molekularpathologisch definierten Subgruppen erlaubt weiterführende Aussagen zur Tumorbiologie und kann ggf. auch mögliche therapeutische Ansatzpunkte identifizieren.
